# Effect of correlations on controllability transition in network control

**DOI:** 10.1038/srep23952

**Published:** 2016-04-11

**Authors:** Sen Nie, Xu-Wen Wang, Bing-Hong Wang, Luo-Luo Jiang

**Affiliations:** 1 Department of Modern Physics, University of Science and Technology of China, Hefei, Anhui 230026, P. R. China; 2 College of Physics and Electronic Information Engineering, Wenzhou University, Wenzhou, Zhejiang 325035, P. R. China; 3 School of Science, Southwest University of Science and Technology, Mianyang, Sichuan 621010, P. R. China

## Abstract

The network control problem has recently attracted an increasing amount of attention, owing to concerns including the avoidance of cascading failures of power-grids and the management of ecological networks. It has been proven that numerical control can be achieved if the number of control inputs exceeds a certain transition point. In the present study, we investigate the effect of degree correlation on the numerical controllability in networks whose topological structures are reconstructed from both real and modeling systems, and we find that the transition point of the number of control inputs depends strongly on the degree correlation in both undirected and directed networks with moderately sparse links. More interestingly, the effect of the degree correlation on the transition point cannot be observed in dense networks for numerical controllability, which contrasts with the corresponding result for structural controllability. In particular, for directed random networks and scale-free networks, the influence of the degree correlation is determined by the types of correlations. Our approach provides an understanding of control problems in complex sparse networks.

One fundamental issue in traditional control theory is controllability, which is defined as the ability for a complex system to be driven from any initial state to any desired state within a finite time by inputting a certain number of control signals^1^. However, it is difficult to determine the minimal number of driver nodes required to achieve full control of a network using traditional control theory. Motivated by this, Liu *et al.* simplified the problem and explored the structural controllability of directed networks, in order to circumvent the need for link weights for applying Kalman’s controllability rank condition^2^. They discovered that the minimal number of driver nodes for a network can be obtained by calculating the maximum matching of the network. Recently, with the development of complex network research, the focus is increasing on problems concerning the controllability of complex dynamical networks^2^,^3^,^4^. In particular, the exact controllability theory offers a more universal approach to the controllability of complex networks with arbitrary structures and link weights, by employing the PBH rank condition^5^,^6^,^7^. It has been proven that the controllability of a network is determined by the maximum geometric multiplicity of the eigenvalues of the adjacent matrix^5^. Recent studies have also focused on control profiles^8^, structural perturbation^9^, control energy^10^, and other important applications relating to controllability^11^,^12^,^13^,^14^,^15^,^16^,^17^,^18^,^19^,^20^. In addition, the controlling of some networks with nonlinear dynamics has also been investigated, such as realistic control in ecological^21^ and biological networks^22^.

Degree correlation is a significant statistical property of complex networks, and characterizes the tendencies of nodes to connect to other nodes with similar in- or out- degrees as themselves. High degree nodes in social networks tend to be connected with other nodes of high degree, while in technological networks and biological networks, nodes of high degree tend to be connected with others of low degree^23^. It has been demonstrated that degree correlation plays an important role in the dynamics of networks^24^,^25^. In particular, degree correlation can produce linear and quadratic dependences on the density of minimal driver nodes in structural controllability^11^. Although structural controllability provides a basis framework for determining the controllability of complex networks, in practice it cannot inform us about the length of the control trajectory, which makes numerical calculations difficult. Considering these problems, the controllability Gramian matrix is an essential tool for numerical calculations. Nevertheless, a system with a well-conditioned Kalman’s controllability matrix may have an ill-conditioned controllability Gramian^26^, which will result in both the control trajectory and control time being too long. Additionally, in practice, the energy consumption cost is also high for controlling a system^22^,^27^,^28^. However, the length of the control trajectory can be reduced and the numerical calculation of the controllability Gramian can be made easier by increasing the number of driver nodes^26^. In analogy with the structural controllability, it is expected that correlation has a significant effect on the length of the control trajectory and the difficulty of achieving successful control in reality. Because the Gramian condition involves the precision of numerical calculations and the success rate of control, we call the controllability calculated using the method of the Gramian condition “numerical controllability”^26^, in order to make the distinction from idealized (structural and exact) notions of controllability (See Methods).

In this study, we investigate the influence of degree correlation on the controllability transition point for topological structures reconstructed from some real and model networks, which is defined as the minimum number of driver nodes required for the success rate of numerical controllability to exceed zero^26^. Our results demonstrate that the transition point displays a non-monotonic dependence on the degree correlation in undirected networks with moderately dense links. For the neutral undirected Erdös-Rényi (ER) and scale-free (SF) networks, it is easier to achieve full control. Meanwhile, all types of degree correlations strongly affect the transition point for directed SF networks. In particular, the transition point depends linearly on the network size in networks with moderately sparse links. All of above results differ greatly from the corresponding results relating to structural controllability^11^.

## Results

### Transition of success control rate for different correlation coefficients

We consider an *N*-dimensional linear time-invariant dynamical system:


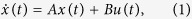


where 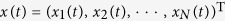 is the state of system at time *t*. *A* is the adjacent matrix which captures the interaction strength between nodes, and *B* is the input matrix which defines how the input signals are connected to the nodes of networks. To eliminate any effect of self-loops on the controllability, we have refrained from using self-loops in our research. Therefore, the diagonal elements of the matrix *A* are all zero, and the driver nodes are defined as nodes controlled by external signals, which are selected randomly in our approach. Because the input vector is written as 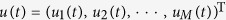, the corresponding minimal energy control input at 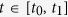 is given by^1^:


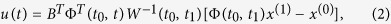


**Figure 1:**
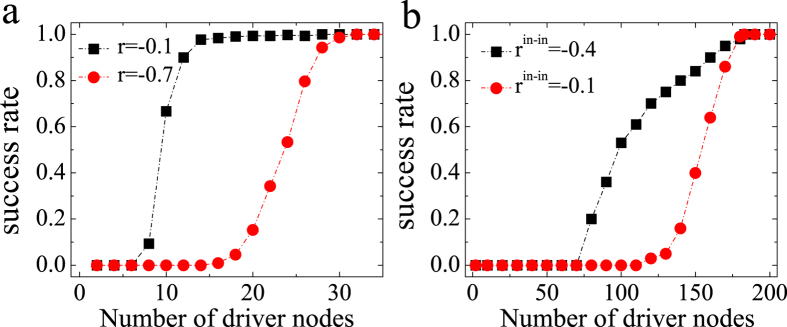
Success rate as a function of the number of driver nodes on topological structures reconstructed from a real network with different degree correlations. (**a**) Karate network; (**b**) Little Rock network. Each data point is an average of 300 independent realizations.

By combining Eqs 1 and 2 with the minimization of the energy 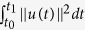, the control trajectory is given by^1^:


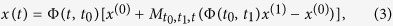


where 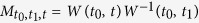, 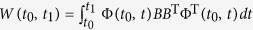 is the controllability Gramian, and 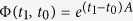
26. Usually, if the controllability matrix 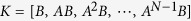 has full rank, then system (*A*, *B*) is considered controllable according to the Kalman’s rank condition^29^, and vice versa. However, with less number of driver nodes, the final state of control *x*(*t*_1_) given by Eq. 3 can not reach the target state *x*^(1)^ in time window [*t*_0_, *t*_1_]. That is, the numerical control fails due to ill-conditioned controllability Gramian. However, the success rate of numerical control depicts transition between zero and one according to an increasing number of driver nodes, as shown in Fig. 1. We denote the minimum rate of driver nodes that corresponds to a success rate firstly greater than zero as the transition point *n*_*d*_ = *N*_*d*_/*N*, where *N*_*d*_ and *N* are the minimum number of driver nodes and the size of nodes in the system respectively. Figure 1 presents, the success control rate for some undirected and directed empirical networks for different correlation coefficients as a function of the number of driver nodes^30^,^31^. We observe that for both undirected and directed networks, the transition points of networked systems with different correlation coefficients are distinct, which demonstrates that numerical control can be influenced by correlation.

### Effect of correlation on numerical controllability and structural controllability

**Figure 2:**
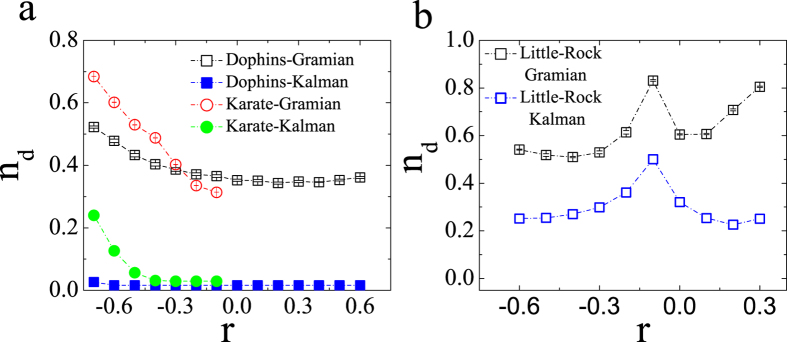
Comparison between the methods of Kalman’s rank condition and Gramian’s condition on topological structures reconstructed from some real networks. (**a**) Dolphins and Karate networks; (**b**) Little Rock network. Each data point is an average of 300 independent realizations, and whiskers in panels (**a**,**b**) show standard deviation (S. D.) of *n*_*d*_.

To distinguish the effects of correlation on structural controllability and numerical controllability, we explore the minimal number of driver nodes as a function of correlation for some empirical networks^30^,^31^,^32^, as illustrated in Fig. 2. We can find that, on the one hand, the number of driver nodes of numerical controllability is always larger than that of structural controllability. On the other hand, for numerical controllability the correlation can produce a monotone linear dependence on *n*_*d*_ for undirected Karate and Dolphins networks, while the *n*_*d*_ only takes maximum and minimum values for different correlations in the directed Little Rock network.

### Numerical transition point for the model network

**Figure 3:**
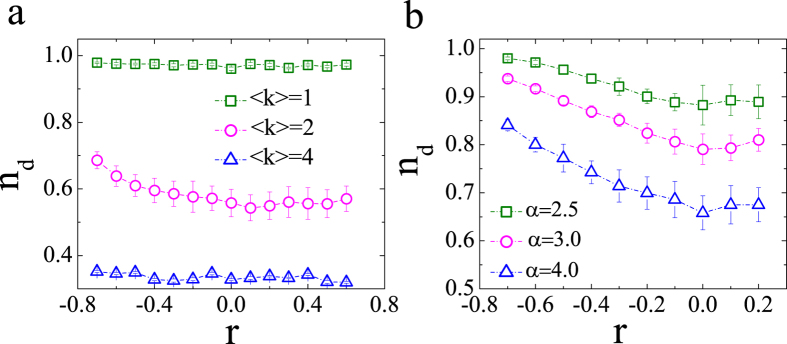
Impacts of degree correlation on the controllability transition point for undirected ER and SF networks (<k> = 2, and power exponent *α* = 2.5) with size *N* = 100. The initial states of the simulations are chosen randomly on the unit sphere centered at the origin and the target states are randomly oriented with *σ* = 10^−2^. Each data point is an average of 300 independent realizations, and values of *n*_*d*_ in panels (**a**,**b**) are shown in the term of “*mean* ± *S.D.*”.

Now, we investigate how degree correlation affects the controllability transition point in undirected ER and SF networks. Simulated annealing is used to vary the degree correlation by link rewiring, while keeping the degree distribution unchanged (for further details, refer to the Methods). Figure 3 illustrates the density of the transition point *n*_*d*_ as a function of degree correlation for undirected networks. Clearly, the curves for the ER networks in Fig. 3(a) can be divided into two categories. The first case is networks with moderately sparse links. *n*_*d*_ displays a quadratic dependence on degree correlation and attains a local minimum at *r* = 0. In this sense, neutral undirected ER networks are the easiest to control. Moreover, disassortative ER networks are more difficult to control than assortative networks. The second category is sparse and dense networks. Here *n*_*d*_ depends weakly on the degree correlation for undirected ER networks. Furthermore, *n*_*d*_ of the undirected SF networks shows the local maximum and minimum, which is completely different from the former situation. Since the degree distributes in the term of *k*^−*α*^ (*α* is defined as the power exponent), we can also find that the larger *α* induces the smaller *n*_*d*_ at the same value of degree correlation. Figure 3 indicates that the correlation has a strong influence on the transition point in undirected networks with moderately sparse links, while the transition point is weakly affected by the correlation in sparse and dense networks. Interestingly, this is different from the corresponding result for structural controllability, where the correlation dramatically impacts the number of driver nodes for dense networks^11^.

**Figure 4:**
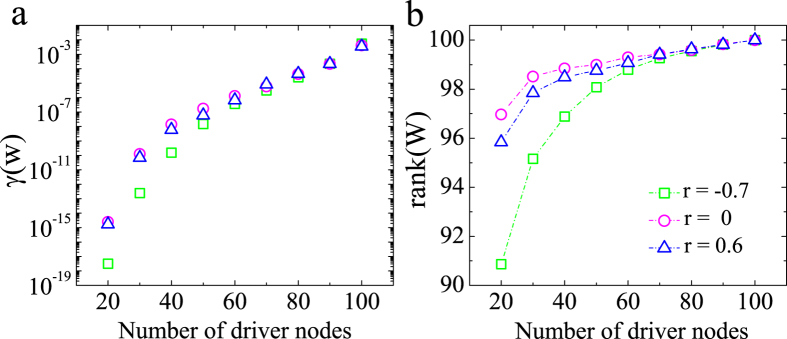
Numerical reciprocal condition number *γ*(*W*) of the controllability Gramian and its rank *rank*(*W*) as functions of diver nodes with different degree correlations for undirected ER networks, with <*k*> = 2. The statistics and parameters not shown are the same as those used in Fig. 3. Each data point is an average of 300 independent realizations.

In order to explore local maximal transition points in undirected networks, we examine the reciprocal condition number *γ*(*W*) and rank(*W*) of the controllability Gramian (*γ*(*W*) is defined as the ratio of the smallest singular value to the largest). Figure 4 depicts the *γ*(*W*) and rank(*W*) as functions of the number of driver nodes for different correlation coefficients *r* in undirected ER networks, with <*k*> = 2. As seen in Fig. 4, *γ*(*W*) and rank(*W*) exhibit rapid growth as the number of driver nodes increases, the controllability Gramian *W* can be well-conditioned, which demonstrates that numerical control can be achieved by increasing the number of inputs. It is known that the smaller the values of *γ*(*W*) and rank(*W*) are, the harder it is to achieve numerical control of the network. By comparing *γ*(*W*) and rank(*W*) with different correlation coefficients, we find that for the same number of driver nodes, the values of *γ*(*W*) and rank(*W*) with *r* = −0.7 are smaller than those with *r* = 0 and *r* = 0.6. Hence, the network with *r* = −0.7 requires a greater number of driver nodes to achieve control, which results in the phenomenon of the maximal transition point in Fig. 3.

**Figure 5:**
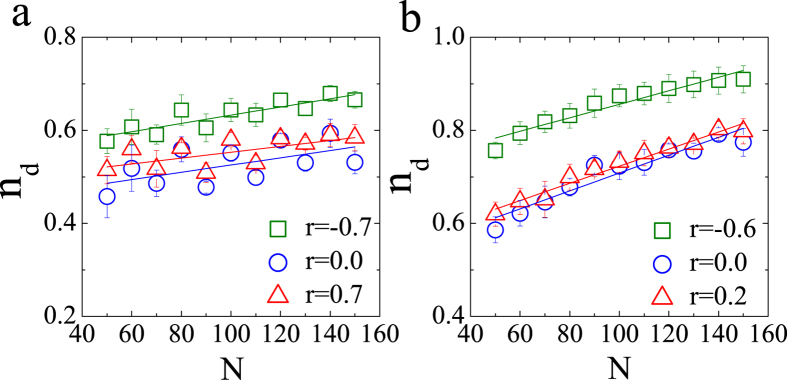
Impacts of degree correlation on the controllability transition point for undirected ER(**a**) and SF(**b**) networks with different network sizes (<*k*> = 2 and *α* = 3). The statistics and parameters not shown are the same as those used in Fig. 3. Each data point is an average of 300 independent realizations, and whiskers in panels (**a**) and (**b**) show standard deviation of *n*_*d*_.

**Figure 6:**
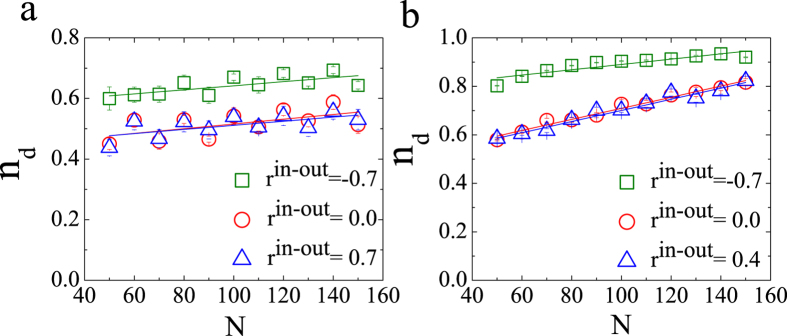
Impacts of degree correlation on the controllability transition point for directed ER (in panel (**a)**) and SF (in panel (**b**)) networks with different network sizes (<*k*> = 2 and *a* = 3). The statistics and parameters not shown are the same as those used in Fig. 3. Each data point is an average of 300 independent realizations, and whiskers in panels (**a**,**b**) show standard deviation of *n*_*d*_.

Furthermore, we explore the controllability transition point as a function of network size for different values of correlation coefficient *r*, as shown in Fig. 5. The transition point *n*_*d*_ for both the undirected ER and SF networks depends linearly on the network size, which is different from the corresponding results for both the structural controllability and the exact controllability. From ref. 5, we know that the minimal number of driver nodes for undirected networks is determined by the algebraic multiplicity of the eigenvalues of the coupling matrix *A*. The increment of the number of driver nodes with network size is extremely small according to the exact controllability theory, while the transition point for numerical control is related linearly to the network size. In other words, the maximal and minimal transition points cannot disappear in large networks. In addition, we study the relationship between the controllability transition point and network size for the directed ER and SF networks, the results of which, are presented in Fig. 6. These results are similar to those for undirected networks. The slope of the linear function presented in Figs 5 and 6 may provide us with a method for estimating the minimal number of driver nodes required for numerical controllability.

**Figure 7:**
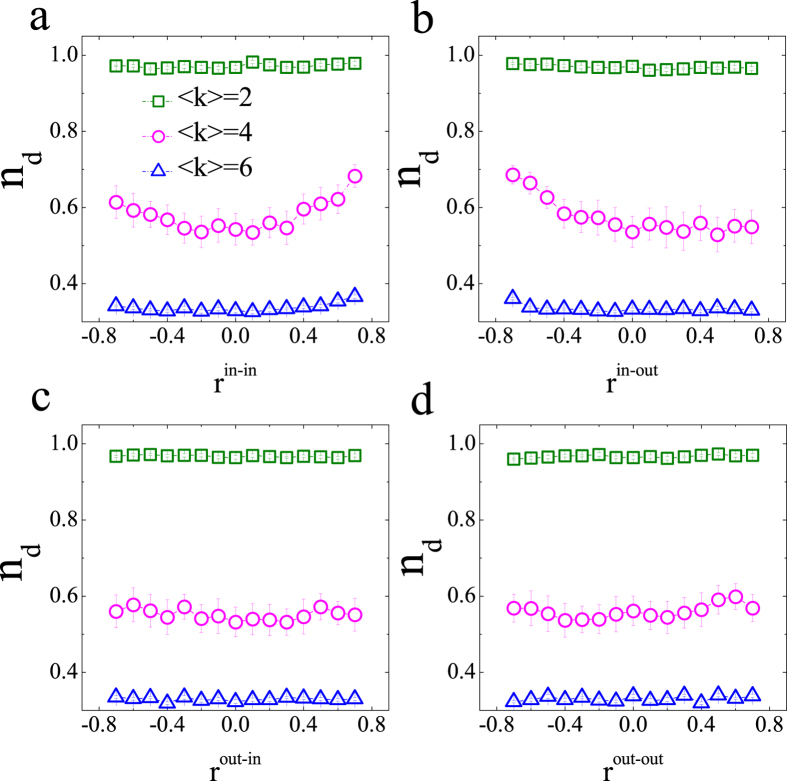
Impact of degree correlation on the controllability transition point for directed ER networks with N = 100. The statistics and parameters not shown are the same as those used in Fig. 3. Each data point is an average of 300 independent realizations, and values of *n*_*d*_ in all panels are shown in the term of “*mean* ± *S.D.*”.

**Figure 8:**
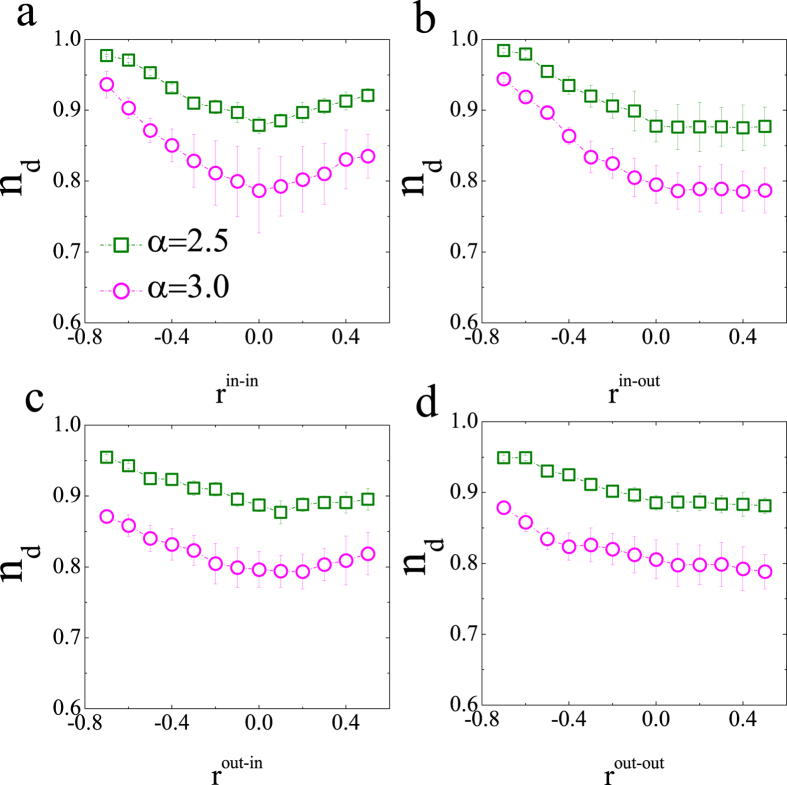
Impact of degree correlation on the controllability transition point for directed SF networks with N = 100 and <*k*> = 4. The statistics and parameters not shown are the same as those used in Fig. 3. Each data point is an average of 300 independent realizations, and values of *n*_*d*_ in all panels are shown in the term of “*mean* ± *S.D.*”.

Finally, it is necessary to explore the case of directed networks. As can be seen in Figs 7 and 8, here *n*_*d*_ exhibits a quadratic dependence on the *r*^in-in^ degree correlation in the ER and SF networks with moderately sparse links. This result is similar to that of undirected ER networks, shown in Fig. 3. However, *n*_*d*_ monotonically decreases according to *r*^in-out^. *r*^out-in^ and *r*^out-out^ have a weak effect on *n*_*d*_. In contrast with the case of ER networks, *r*^in-out^, *r*^out-in^ and *r*^out-out^ all cause *n*_*d*_ to decrease in SF networks. To summarize, the most striking differences between the above results and the corresponding conclusions for the structural controllability lies on that the transition point depends strongly on the degree correlation in networks with moderately sparse links. In contrast, it cannot be affected by the degree correlation in sparse and dense networks, which is completely different from those results in structural controllability that the minimal number of driver nodes can be significantly affected for dense networks^11^. In addition, the value of the transition point in directed networks is larger than that in undirected networks, which indicates the difference in controllability between directed and undirected networks.

It is worth mentioning that the driver nodes for all simulations are selected randomly. Concerning the structural controllability of directed networks, the value of the minimal number of driver nodes depends sensitively on the importance of the selected driver nodes in the hierarchical structure^13^. In this paper, we also choose driver nodes according to the differences in their importance, such as the degree and betweenness centrality. However, these selection methods cannot efficiently affect the value of the transition point.

## Discussion

Previous studies have offered effective tools for approaching the issue of controllability in complex networks based on the Kalman and PBH rank conditions^2^,^5^, to examine whether a system can be driven from any initial state to any desired state within finite time. However, in practice the controllability Gramian becomes an essential factor in obtaining the length of the control trajectory^26^. In general, the Kalman rank condition is equivalent to the Gramian’s rank condition. Nonetheless, a system with a well-conditioned Kalman’s controllability matrix may have an ill-conditioned controllability Gramian^26^. In this case, the length of the control trajectory will be too long and the cost will be too high. By increasing the number of driver nodes, the controllability Gramian becomes well-conditioned, and numerical control can be achieved^26^. Motivated by the fact that the degree correlation influences the numerical calculation of the Gramian matrix, we have examined how the degree correlation affects the controllability transition point of networks, and illustrated the difficulty of achieving numerical control of networks with varied degree correlations.

We performed numerical simulations in order to reveal the effect of degree correlation on the transition point in undirected and directed networks. We found that the neutral undirected ER and SF networks are the easiest to control, and the transition point can only be influenced by the correlation in both undirected and directed networks with moderately sparse links. For sparse networks, it is necessary to control almost all of the nodes, while for dense networks, a lower fraction of driver nodes can ensure that numerical control is successful. Therefore, a varying correlation cannot affect the transition points in either sparse or dense networks. In particular, the transition point depends linearly on the network size based on the minimal energy control. These results are dramatically different from those relating to structural and exact controllability. It is worth mentioning that the dynamics on the topological structures of both real and model networks is only linearly time-invariant, while the dynamics of real systems may be time-varying. In the future work, we expect to further study the relationship between controllability and the structure of complex networks based on the Gramian matrix.

## Methods

### Controllability of complex networks

For a *N*-dimensional linear time-invariant dynamical system, 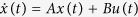, it is considered as controllable if controllability condition can be satisfied. According to different controllability conditions suitable for different networks and calculations, the controllability of complex networks can be divided as follows:

1. Structural controllability: In the Kalman’s rank condition^29^, the system is controllable when the following Eq. 4 is satisfied.
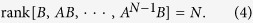
However, it is difficult to calculate controllability matrix (the left part of Eq. 4) if the weights of some matrix elements in matrix *A* are unknown. To solve the problem, an effective approach is to choose the non-zero weights in matrix *A* and matrix *B* which satisfy Eq. 4, then the system is structural controllable accordingly. Furthermore, the minimal number of driver nodes which is needed to fully control the directed networks can be calculated by the maximum matching^2^.
2. Exact controllability: According to the PBH rank condition^6^, authors of ref. 5 have proven that the minimal number of driver nodes of controllability for the network with arbitrary structure and link weights is given by the following Eq. 5:
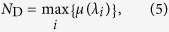
where *μ*(*λ*_*i*_) is the geometric multiplicity of the distinct eigenvalue *λ*_*i*_ for the adjacent matrix *A*. This general method based on the multiplicity of eigenvalues is called exact controllability theory.
3. Numerical controllability: According to the Gramian condition^1^, the system is controllable when the Gramian matrix 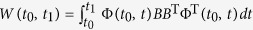 is well-conditioned. Because the Gramian matrix refers to the numerical calculation of control trajectory and energy consumption, the system is considered as numerical controllable if it satisfies the Gramian condition. However, due to numerical calculation, the structural controllable or exact controllable system probably has the ill-conditioned Gramian matrix. Therefore, the minimal number of driver nodes may not be the same for different controllability criterions.

### Degree correlation

Each node in a network has an in-degree *k*_in_ and an out-degree *k*_out_, and we ensure that no self-loops exist in the networks. The degree correlation measures the tendencies of nodes to connect with other nodes that have similar in- and out- degrees as themselves. This can be quantified by using the Pearson coefficient^23^:


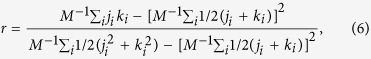


where 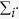 sums over all edges, *j*_*i*_ and *k*_*i*_ are the degrees of nodes which are connected to the *i*th edge, and *M* is the total number of edges. For undirected networks, *j* and *k* are the degrees of two nodes belonging to edge *i*. A positive value of *r* indicates the assortative network while the negative value of *r* characterizes a disassortative network.

### Network construction and calculation of transition point

Simulated annealing is used to obtain networks with the desired degree correlation, by rewiring links while leaving the in- and out- degrees unchanged. It is worth mentioning that self-loops are not permitted during the rewiring operation, or in the original network. We set the desired degree correlation r* and energy *E*(*r*) = |*r* − r*| by carrying out the following steps to minimize the energy. (I) Initialize the parameters: temperature *T* and *E*(*r*); (II) Choose two edges with an equal probability; (III) Rewire the two edges while leaving the in- and out- degrees unchanged, and calculate the energy of the resulting network *E*(*r*′); (IV) The new configuration can be accepted with probability


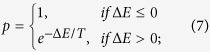


(V) Calculate 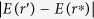, and if it is smaller than the defined value (here, we set the defined value is 0.01) then stop, else repeat from step (2) and gradually decrease *T*.

In addition to the real network structures of some empirical networks^30^,^31^,^32^, models of Erdös-Rényi (ER) and Scale-free (SF) networks are also used to study the effects of correlations on the controllability transition. The edges are assigned weights, drawn from a uniform distribution in [−1, 1]. *B* is a diagonal matrix, whose diagonal elements *B*_*ii*_ are assigned 1 when the *i*th node is a driver node, and a fraction of nodes *f* are randomly chosen as driver nodes at each independent realization. For an *N*-dimensional linear time-invariant dynamical system, the control trajectory with minimized energy at *t* ∈ [*t*_0_, *t*_1_] is given by^1^:


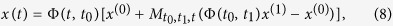


where the initial states of the simulations *x*^(0)^ are chosen randomly on the unit sphere centered at the origin, and the target states *x*^(1)^ are randomly oriented to be *σ* = 10^−2^ apart, where *σ* is the distance from the initial state to the target state. We consider the numerical control to be successful if the distance between the calculated state *x*(*t*_1_) from Eq. 3 and the target state *x*^(1)^ is less than *η* in the time window *t*_0_ ≤ *t* ≤ *t*_1_ for *t*_0_ = 0, *t*_1_ = 1 and 
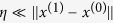
, with *η* = 10^−6^. Then, the corresponding rate of driver nodes is defined as the transition point *n*_*d*_ (*n*_*d*_ = *N*_*d*_/*N*, where *N*_*d*_ and *N* are the minimum number of driver nodes and the size of nodes in the system respectively). In this fashion, we compute the transition points in different networks with different values of degree correlation *r*.

## Additional Information

**How to cite this article**: Nie, S. *et al.* Effect of correlations on controllability transition in network control. *Sci. Rep.* **6**, 23952; doi: 10.1038/srep23952 (2016).
